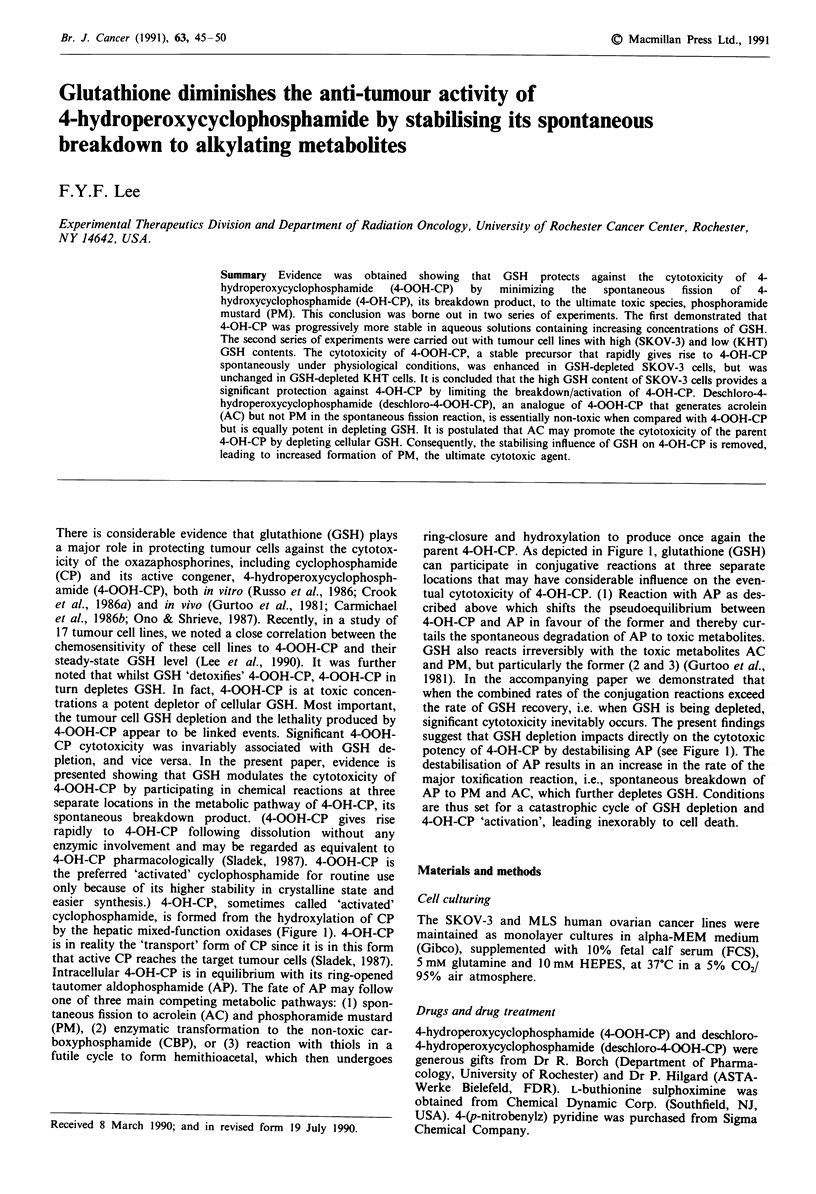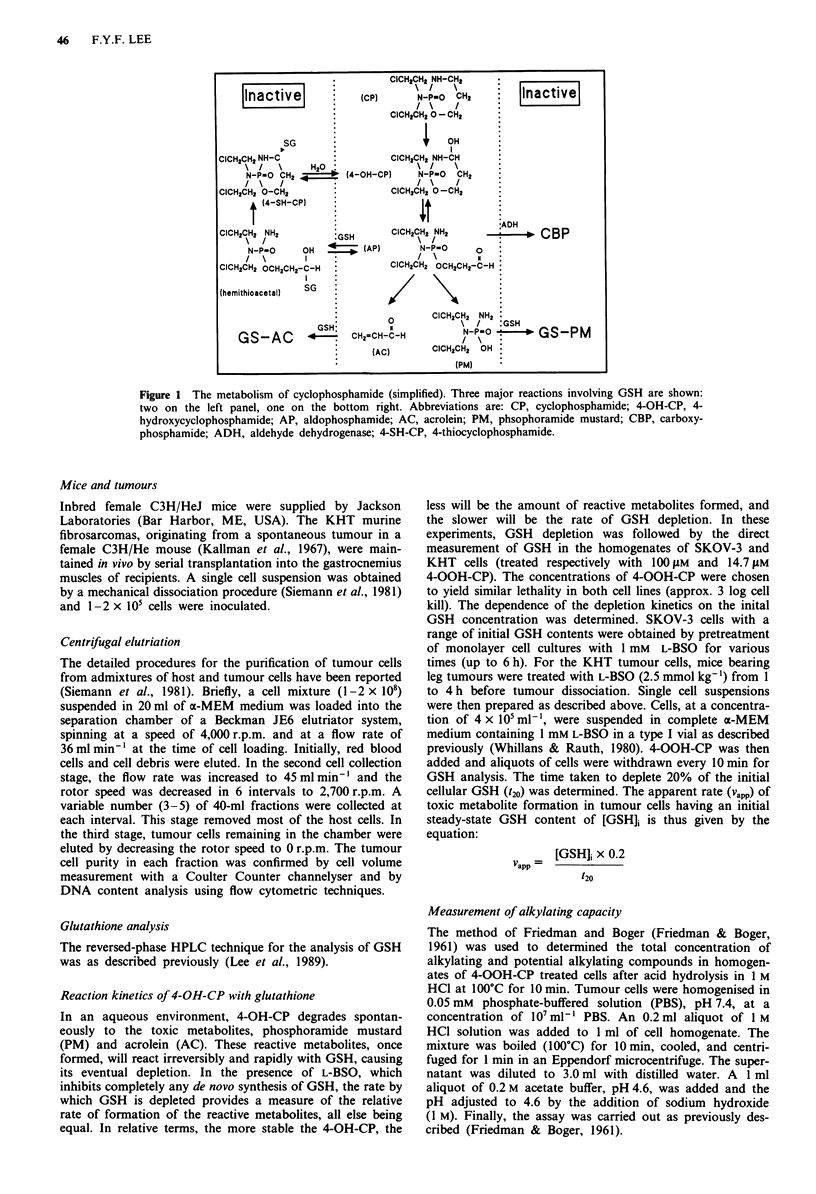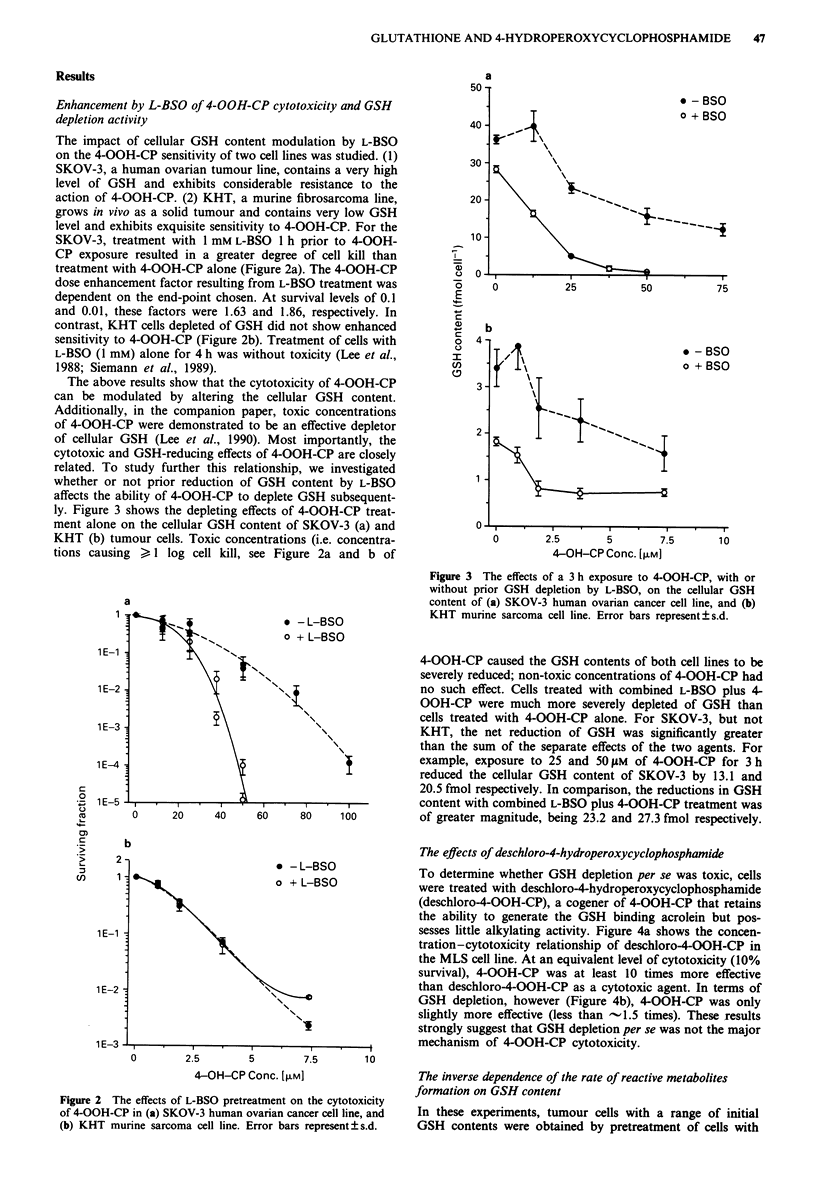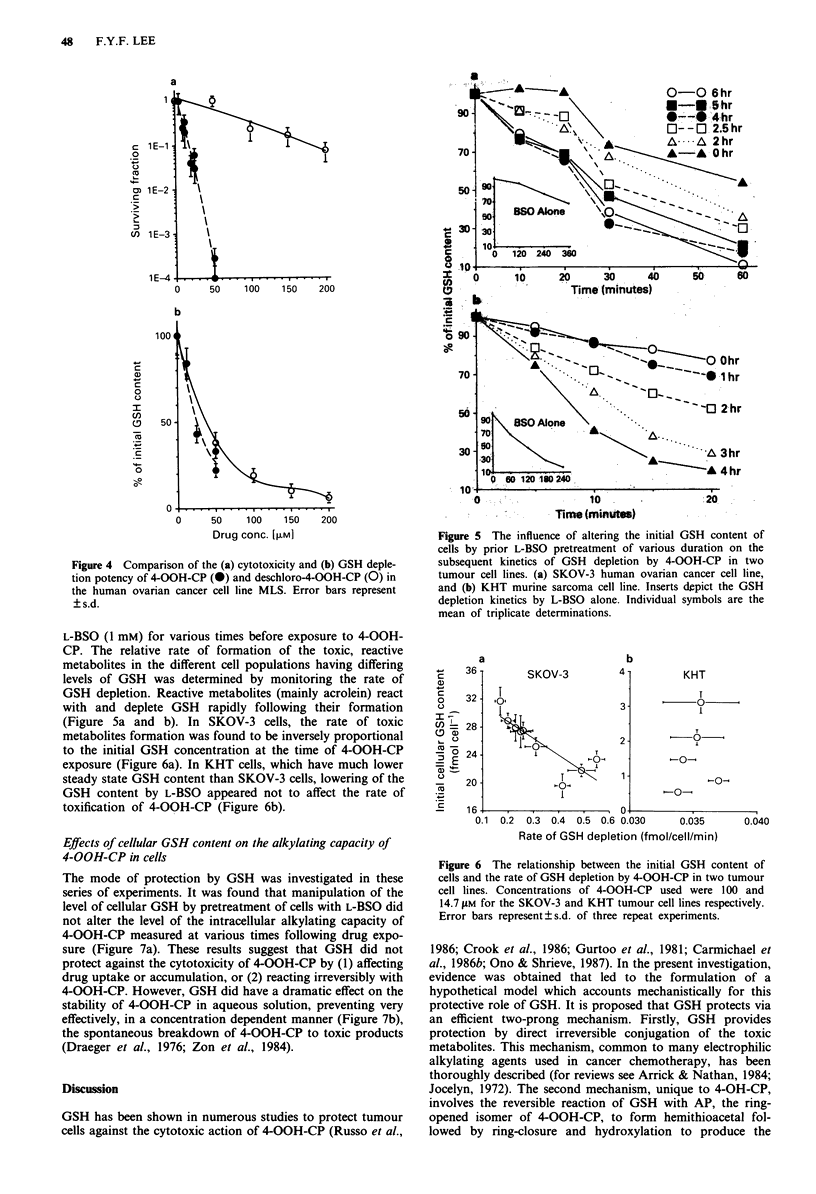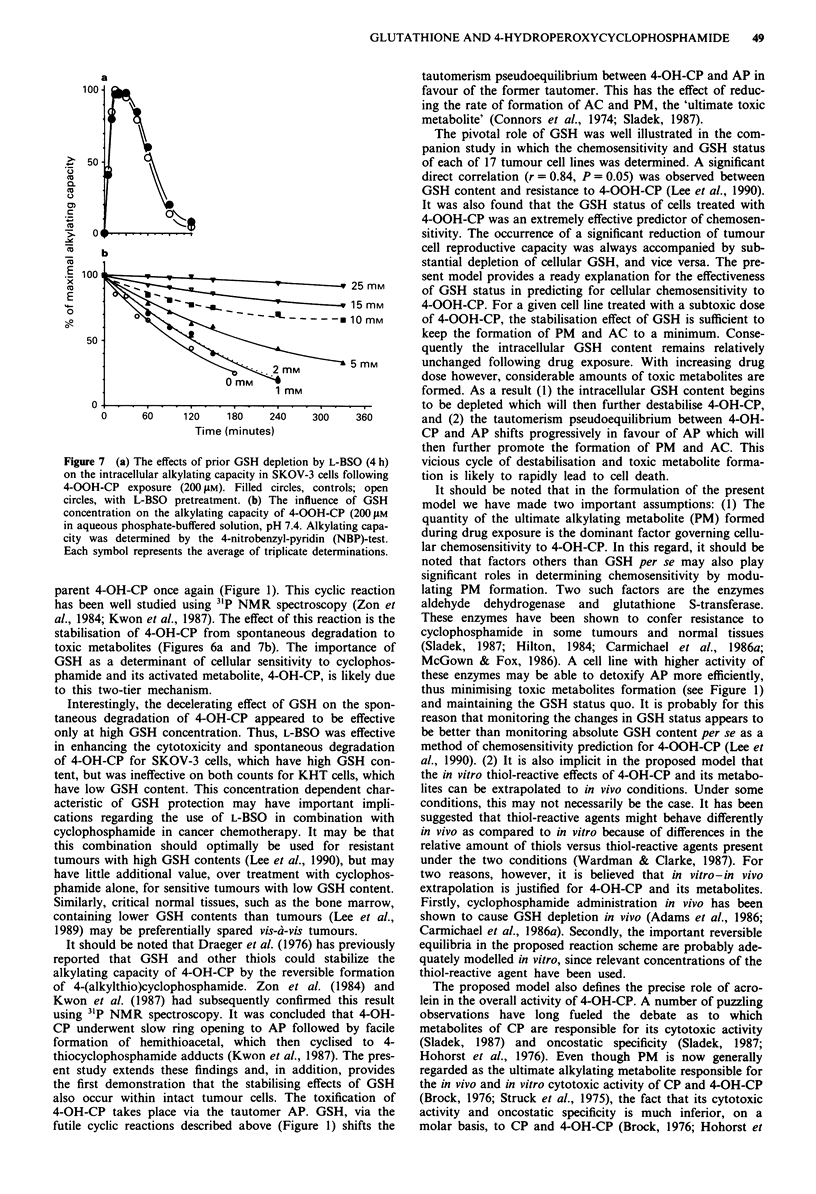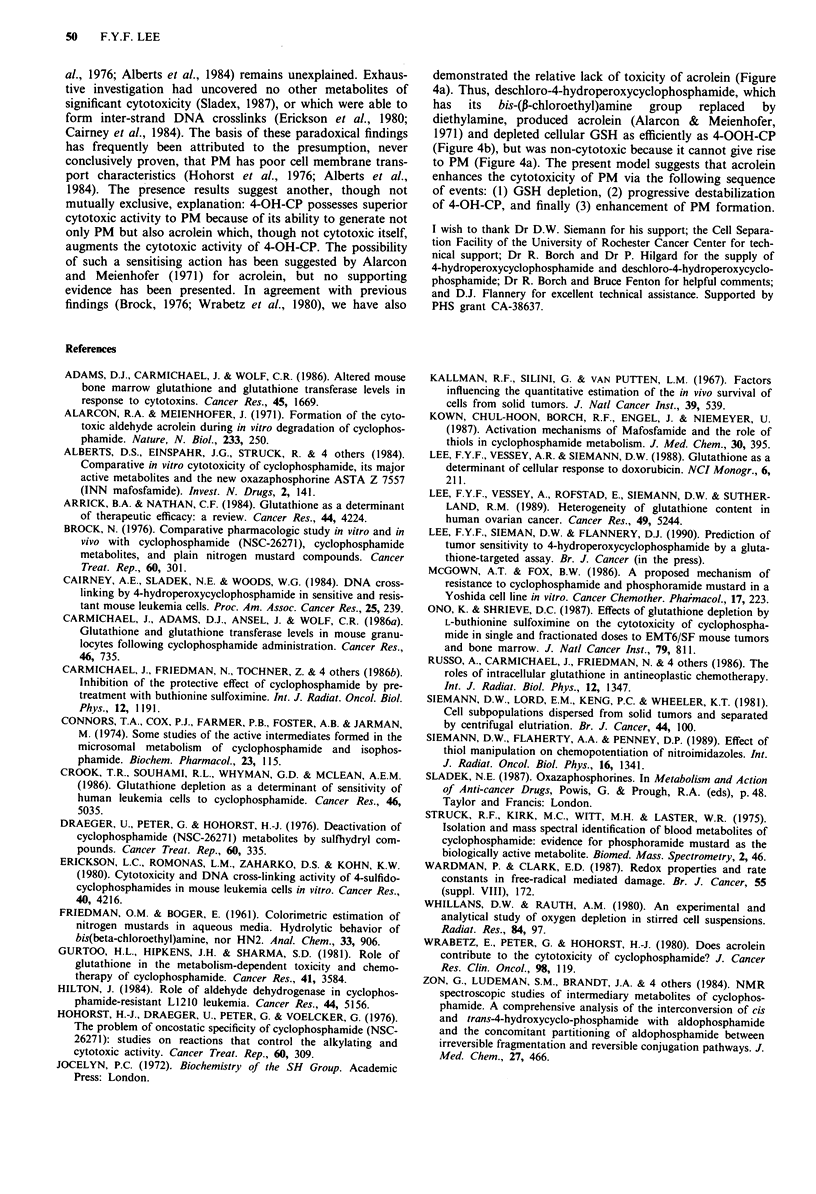# Glutathione diminishes the anti-tumour activity of 4-hydroperoxycyclophosphamide by stabilising its spontaneous breakdown to alkylating metabolites.

**DOI:** 10.1038/bjc.1991.10

**Published:** 1991-01

**Authors:** F. Y. Lee

**Affiliations:** Experimental Therapeutics Division, University of Rochester Cancer Center, NY 14642.

## Abstract

Evidence was obtained showing that GSH protects against the cytotoxicity of 4-hydroperoxycyclophosphamide (4-OOH-CP) by minimizing the spontaneous fission of 4-hydroxycyclophosphamide (4-OH-CP), its breakdown product, to the ultimate toxic species, phosphoramide mustard (PM). This conclusion was borne out in two series of experiments. The first demonstrated that 4-OH-CP was progressively more stable in aqueous solutions containing increasing concentrations of GSH. The second series of experiments were carried out with tumour cell lines with high (SKOV-3) and low (KHT) GSH contents. The cytotoxicity of 4-OOH-CP, a stable precursor that rapidly gives rise to 4-OH-CP spontaneously under physiological conditions, was enhanced in GSH-depleted SKOV-3 cells, but was unchanged in GSH-depleted KHT cells. It is concluded that the high GSH content of SKOV-3 cells provides a significant protection against 4-OH-CP by limiting the breakdown/activation of 4-OH-CP. Deschloro-4-hydroperoxycyclophosphamide (deschloro-4-OOH-CP), an analogue of 4-OOH-CP that generates acrolein (AC) but not PM in the spontaneous fission reaction, is essentially non-toxic when compared with 4-OOH-CP but is equally potent in depleting GSH. It is postulated that AC may promote the cytotoxicity of the parent 4-OH-CP by depleting cellular GSH. Consequently, the stabilising influence of GSH on 4-OH-CP is removed, leading to increased formation of PM, the ultimate cytotoxic agent.


 © Macmillan Press Ltd., 1991

### Glutathione diminishes the anti-tumour activity of 4-hydroperoxycyclophosphamide by stabilising its spontaneous breakdown to alkylating metabolites

F.Y.F. Lee

*Experimental Therapeutics Division and Department of Radiation Oncology, University of Rochester Cancer Center, Rochester, NY 14642, USA.*

**Summary** Evidence was obtained showing that GSH protects against the cytotoxicity of 4-hydroperoxycyclophosphamide (4-OOH-CP) by minimizing the spontaneous fission of 4-hydroxycyclophosphamide (4-OH-CP), its breakdown product, to the ultimate toxic species, phosphoramide mustard (PM). This conclusion was borne out in two series of experiments. The first demonstrated that 4-OH-CP was progressively more stable in aqueous solutions containing increasing concentrations of GSH. The second series of experiments were carried out with tumour cell lines with high (SKOV-3) and low (KHT) GSH contents. The cytotoxicity of 4-OOH-CP, a stable precursor that rapidly gives rise to 4-OH-CP spontaneously under physiological conditions, was enhanced in GSH-depleted SKOV-3 cells, but was unchanged in GSH-depleted KHT cells. It is concluded that the high GSH content of SKOV-3 cells provides a significant protection against 4-OH-CP by limiting the breakdown/activation of 4-OH-CP. Deschloro-4-hydroperoxycyclophosphamide (deschloro-4-OOH-CP), an analogue of 4-OOH-CP that generates acrolein (AC) but not PM in the spontaneous fission reaction, is essentially non-toxic when compared with 4-OOH-CP but is equally potent in depleting GSH. It is postulated that AC may promote the cytotoxicity of the parent 4-OH-CP by depleting cellular GSH. Consequently, the stabilising influence of GSH on 4-OH-CP is removed, leading to increased formation of PM, the ultimate cytotoxic agent.

There is considerable evidence that glutathione (GSH) plays a major role in protecting tumour cells against the cytotoxicity of the oxazaphosphorines, including cyclophosphamide (CP) and its active congener, 4-hydroperoxycyclophosphamide (4-OOH-CP), both *in vitro* (Russo *et al.*, 1986; Crook *et al.*, 1986*a*) and *in vivo* (Gurtoo *et al.*, 1981; Carmichael *et al.*, 1986*b*; Ono & Shrieve, 1987). Recently, in a study of 17 tumour cell lines, we noted a close correlation between the chemosensitivity of these cell lines to 4-OOH-CP and their steady-state GSH level (Lee *et al.*, 1990). It was further noted that whilst GSH 'detoxifies' 4-OOH-CP, 4-OOH-CP in turn depletes GSH. In fact, 4-OOH-CP is at toxic concentrations a potent depletor of cellular GSH. Most important, the tumour cell GSH depletion and the lethality produced by 4-OOH-CP appear to be linked events. Significant 4-OOH-CP cytotoxicity was invariably associated with GSH depletion, and vice versa. In the present paper, evidence is presented showing that GSH modulates the cytotoxicity of 4-OOH-CP by participating in chemical reactions at three separate locations in the metabolic pathway of 4-OH-CP, its spontaneous breakdown product. (4-OOH-CP gives rise rapidly to 4-OH-CP following dissolution without any enzymic involvement and may be regarded as equivalent to 4-OH-CP pharmacologically (Sladek, 1987). 4-OOH-CP is the preferred 'activated' cyclophosphamide for routine use only because of its higher stability in crystalline state and easier synthesis.) 4-OH-CP, sometimes called 'activated' cyclophosphamide, is formed from the hydroxylation of CP by the hepatic mixed-function oxidases (Figure 1). 4-OH-CP is in reality the 'transport' form of CP since it is in this form that active CP reaches the target tumour cells (Sladek, 1987). Intracellular 4-OH-CP is in equilibrium with its ring-opened tautomer aldophosphamide (AP). The fate of AP may follow one of three main competing metabolic pathways: (1) spontaneous fission to acrolein (AC) and phosphoramide mustard (PM), (2) enzymatic transformation to the non-toxic carboxyphosphamide (CBP), or (3) reaction with thiols in a futile cycle to form hemithioacetal, which then undergoes ring-closure and hydroxylation to produce once again the parent 4-OH-CP. As depicted in Figure 1, glutathione (GSH) can participate in conjugative reactions at three separate locations that may have considerable influence on the eventual cytotoxicity of 4-OH-CP. (1) Reaction with AP as described above which shifts the pseudoequilibrium between 4-OH-CP and AP in favour of the former and thereby curtails the spontaneous degradation of AP to toxic metabolites. GSH also reacts irreversibly with the toxic metabolites AC and PM, but particularly the former (2 and 3) (Gurtoo *et al.*, 1981). In the accompanying paper we demonstrated that when the combined rates of the conjugation reactions exceed the rate of GSH recovery, i.e. when GSH is being depleted, significant cytotoxicity inevitably occurs. The present findings suggest that GSH depletion impacts directly on the cytotoxic potency of 4-OH-CP by destabilising AP (see Figure 1). The destabilisation of AP results in an increase in the rate of the major toxification reaction, i.e., spontaneous breakdown of AP to PM and AC, which further depletes GSH. Conditions are thus set for a catastrophic cycle of GSH depletion and 4-OH-CP 'activation', leading inexorably to cell death.

### Materials and methods

### *Cell culturing*

The SKOV-3 and MLS human ovarian cancer lines were maintained as monolayer cultures in alpha-MEM medium (Gibco), supplemented with 10% fetal calf serum (FCS), 5 mM glutamine and 10 mM HEPES, at 37°C in a 5% $CO_2$/ 95% air atmosphere.

### *Drugs and drug treatment*

4-hydroperoxycyclophosphamide (4-OOH-CP) and deschloro-4-hydroperoxycyclophosphamide (deschloro-4-OOH-CP) were generous gifts from Dr R. Borch (Department of Pharmacology, University of Rochester) and Dr P. Hilgard (ASTA-Werke Bielefeld, FDR). L-buthionine sulphoximine was obtained from Chemical Dynamic Corp. (Southfield, NJ, USA). 4-(*p*-nitrobenylz) pyridine was purchased from Sigma Chemical Company.

Received 8 March 1990; and in revised form 19 July 1990.

**Figure 1** The metabolism of cyclophosphamide (simplified). Three major reactions involving GSH are shown: two on the left panel, one on the bottom right. Abbreviations are: CP, cyclophosphamide; 4-OH-CP, 4-hydroxycyclophosphamide; AP, aldophosphamide; AC, acrolein; PM, phsophoramide mustard; CBP, carboxyphosphamide; ADH, aldehyde dehydrogenase; 4-SH-CP, 4-thiocyclophosphamide.

*Mice and tumours*

Inbred female C3H/HeJ mice were supplied by Jackson Laboratories (Bar Harbor, ME, USA). The KHT murine fibrosarcomas, originating from a spontaneous tumour in a female C3H/He mouse (Kallman *et al.*, 1967), were maintained *in vivo* by serial transplantation into the gastrocnemius muscles of recipients. A single cell suspension was obtained by a mechanical dissociation procedure (Siemann *et al.*, 1981) and $1-2 \times 10^5$ cells were inoculated.

*Centrifugal elutriation*

The detailed procedures for the purification of tumour cells from admixtures of host and tumour cells have been reported (Siemann *et al.*, 1981). Briefly, a cell mixture ($1-2 \times 10^8$) suspended in 20 ml of α-MEM medium was loaded into the separation chamber of a Beckman JE6 elutriator system, spinning at a speed of 4,000 r.p.m. and at a flow rate of 36 ml $min^{-1}$ at the time of cell loading. Initially, red blood cells and cell debris were eluted. In the second cell collection stage, the flow rate was increased to 45 ml $min^{-1}$ and the rotor speed was decreased in 6 intervals to 2,700 r.p.m. A variable number (3–5) of 40-ml fractions were collected at each interval. This stage removed most of the host cells. In the third stage, tumour cells remaining in the chamber were eluted by decreasing the rotor speed to 0 r.p.m. The tumour cell purity in each fraction was confirmed by cell volume measurement with a Coulter Counter channelyser and by DNA content analysis using flow cytometric techniques.

*Glutathione analysis*

The reversed-phase HPLC technique for the analysis of GSH was as described previously (Lee *et al.*, 1989).

*Reaction kinetics of 4-OH-CP with glutathione*

In an aqueous environment, 4-OH-CP degrades spontaneously to the toxic metabolites, phosphoramide mustard (PM) and acrolein (AC). These reactive metabolites, once formed, will react irreversibly and rapidly with GSH, causing its eventual depletion. In the presence of L-BSO, which inhibits completely any *de novo* synthesis of GSH, the rate by which GSH is depleted provides a measure of the relative rate of formation of the reactive metabolites, all else being equal. In relative terms, the more stable the 4-OH-CP, the less will be the amount of reactive metabolites formed, and the slower will be the rate of GSH depletion. In these experiments, GSH depletion was followed by the direct measurement of GSH in the homogenates of SKOV-3 and KHT cells (treated respectively with 100 μM and 14.7 μM 4-OOH-CP). The concentrations of 4-OOH-CP were chosen to yield similar lethality in both cell lines (approx. 3 log cell kill). The dependence of the depletion kinetics on the inital GSH concentration was determined. SKOV-3 cells with a range of initial GSH contents were obtained by pretreatment of monolayer cell cultures with 1 mM L-BSO for various times (up to 6 h). For the KHT tumour cells, mice bearing leg tumours were treated with L-BSO (2.5 mmol $kg^{-1}$) from 1 to 4 h before tumour dissociation. Single cell suspensions were then prepared as described above. Cells, at a concentration of $4 \times 10^5$ $ml^{-1}$, were suspended in complete α-MEM medium containing 1 mM L-BSO in a type I vial as described previously (Whillans & Rauth, 1980). 4-OOH-CP was then added and aliquots of cells were withdrawn every 10 min for GSH analysis. The time taken to deplete 20% of the initial cellular GSH ($t_{20}$) was determined. The apparent rate ($v_{app}$) of toxic metabolite formation in tumour cells having an initial steady-state GSH content of $[GSH]_i$ is thus given by the equation:

$$v_{app} = \frac{[GSH]_i \times 0.2}{t_{20}}$$

*Measurement of alkylating capacity*

The method of Friedman and Boger (Friedman & Boger, 1961) was used to determined the total concentration of alkylating and potential alkylating compounds in homogenates of 4-OOH-CP treated cells after acid hydrolysis in 1 M HCl at 100°C for 10 min. Tumour cells were homogenised in 0.05 mM phosphate-buffered solution (PBS), pH 7.4, at a concentration of $10^7$ $ml^{-1}$ PBS. An 0.2 ml aliquot of 1 M HCl solution was added to 1 ml of cell homogenate. The mixture was boiled (100°C) for 10 min, cooled, and centrifuged for 1 min in an Eppendorf microcentrifuge. The supernatant was diluted to 3.0 ml with distilled water. A 1 ml aliquot of 0.2 M acetate buffer, pH 4.6, was added and the pH adjusted to 4.6 by the addition of sodium hydroxide (1 M). Finally, the assay was carried out as previously described (Friedman & Boger, 1961).

### Results

### *Enhancement by L-BSO of 4-OOH-CP cytotoxicity and GSH depletion activity*

The impact of cellular GSH content modulation by L-BSO on the 4-OOH-CP sensitivity of two cell lines was studied. (1) SKOV-3, a human ovarian tumour line, contains a very high level of GSH and exhibits considerable resistance to the action of 4-OOH-CP. (2) KHT, a murine fibrosarcoma line, grows *in vivo* as a solid tumour and contains very low GSH level and exhibits exquisite sensitivity to 4-OOH-CP. For the SKOV-3, treatment with 1 mM L-BSO 1 h prior to 4-OOH-CP exposure resulted in a greater degree of cell kill than treatment with 4-OOH-CP alone (Figure 2a). The 4-OOH-CP dose enhancement factor resulting from L-BSO treatment was dependent on the end-point chosen. At survival levels of 0.1 and 0.01, these factors were 1.63 and 1.86, respectively. In contrast, KHT cells depleted of GSH did not show enhanced sensitivity to 4-OOH-CP (Figure 2b). Treatment of cells with L-BSO (1 mM) alone for 4 h was without toxicity (Lee *et al.*, 1988; Siemann *et al.*, 1989).

The above results show that the cytotoxicity of 4-OOH-CP can be modulated by altering the cellular GSH content. Additionally, in the companion paper, toxic concentrations of 4-OOH-CP were demonstrated to be an effective depletor of cellular GSH (Lee *et al.*, 1990). Most importantly, the cytotoxic and GSH-reducing effects of 4-OOH-CP are closely related. To study further this relationship, we investigated whether or not prior reduction of GSH content by L-BSO affects the ability of 4-OOH-CP to deplete GSH subsequently. Figure 3 shows the depleting effects of 4-OOH-CP treatment alone on the cellular GSH content of SKOV-3 (a) and KHT (b) tumour cells. Toxic concentrations (i.e. concentrations causing $\geqslant 1$ log cell kill, see Figure 2a and b of 4-OOH-CP caused the GSH contents of both cell lines to be severely reduced; non-toxic concentrations of 4-OOH-CP had no such effect. Cells treated with combined L-BSO plus 4-OOH-CP were much more severely depleted of GSH than cells treated with 4-OOH-CP alone. For SKOV-3, but not KHT, the net reduction of GSH was significantly greater than the sum of the separate effects of the two agents. For example, exposure to 25 and 50 µM of 4-OOH-CP for 3 h reduced the cellular GSH content of SKOV-3 by 13.1 and 20.5 fmol respectively. In comparison, the reductions in GSH content with combined L-BSO plus 4-OOH-CP treatment was of greater magnitude, being 23.2 and 27.3 fmol respectively.

**Figure 2** The effects of L-BSO pretreatment on the cytotoxicity of 4-OOH-CP in (a) SKOV-3 human ovarian cancer cell line, and (b) KHT murine sarcoma cell line. Error bars represent ± s.d.

**Figure 3** The effects of a 3 h exposure to 4-OOH-CP, with or without prior GSH depletion by L-BSO, on the cellular GSH content of (a) SKOV-3 human ovarian cancer cell line, and (b) KHT murine sarcoma cell line. Error bars represent ± s.d.

### *The effects of deschloro-4-hydroperoxycyclophosphamide*

To determine whether GSH depletion *per se* was toxic, cells were treated with deschloro-4-hydroperoxycyclophosphamide (deschloro-4-OOH-CP), a cogener of 4-OOH-CP that retains the ability to generate the GSH binding acrolein but possesses little alkylating activity. Figure 4a shows the concentration–cytotoxicity relationship of deschloro-4-OOH-CP in the MLS cell line. At an equivalent level of cytotoxicity (10% survival), 4-OOH-CP was at least 10 times more effective than deschloro-4-OOH-CP as a cytotoxic agent. In terms of GSH depletion, however (Figure 4b), 4-OOH-CP was only slightly more effective (less than ~1.5 times). These results strongly suggest that GSH depletion *per se* was not the major mechanism of 4-OOH-CP cytotoxicity.

### *The inverse dependence of the rate of reactive metabolites formation on GSH content*

In these experiments, tumour cells with a range of initial GSH contents were obtained by pretreatment of cells with

Figure 4 Comparison of the (a) cytotoxicity and (b) GSH depletion potency of 4-OOH-CP (●) and deschloro-4-OOH-CP (O) in the human ovarian cancer cell line MLS. Error bars represent ±s.d.

L-BSO (1 mM) for various times before exposure to 4-OOH-CP. The relative rate of formation of the toxic, reactive metabolites in the different cell populations having differing levels of GSH was determined by monitoring the rate of GSH depletion. Reactive metabolites (mainly acrolein) react with and deplete GSH rapidly following their formation (Figure 5a and b). In SKOV-3 cells, the rate of toxic metabolites formation was found to be inversely proportional to the initial GSH concentration at the time of 4-OOH-CP exposure (Figure 6a). In KHT cells, which have much lower steady state GSH content than SKOV-3 cells, lowering of the GSH content by L-BSO appeared not to affect the rate of toxification of 4-OOH-CP (Figure 6b).

*Effects of cellular GSH content on the alkylating capacity of 4-OOH-CP in cells*

The mode of protection by GSH was investigated in these series of experiments. It was found that manipulation of the level of cellular GSH by pretreatment of cells with L-BSO did not alter the level of the intracellular alkylating capacity of 4-OOH-CP measured at various times following drug exposure (Figure 7a). These results suggest that GSH did not protect against the cytotoxicity of 4-OOH-CP by (1) affecting drug uptake or accumulation, or (2) reacting irreversibly with 4-OOH-CP. However, GSH did have a dramatic effect on the stability of 4-OOH-CP in aqueous solution, preventing very effectively, in a concentration dependent manner (Figure 7b), the spontaneous breakdown of 4-OOH-CP to toxic products (Draeger *et al.*, 1976; Zon *et al.*, 1984).

### Discussion

GSH has been shown in numerous studies to protect tumour cells against the cytotoxic action of 4-OOH-CP (Russo *et al.*, 1986; Crook *et al.*, 1986; Gurtoo *et al.*, 1981; Carmichael *et al.*, 1986*b*; Ono & Shrieve, 1987). In the present investigation, evidence was obtained that led to the formulation of a hypothetical model which accounts mechanistically for this protective role of GSH. It is proposed that GSH protects via an efficient two-prong mechanism. Firstly, GSH provides protection by direct irreversible conjugation of the toxic metabolites. This mechanism, common to many electrophilic alkylating agents used in cancer chemotherapy, has been thoroughly described (for reviews see Arrick & Nathan, 1984; Jocelyn, 1972). The second mechanism, unique to 4-OH-CP, involves the reversible reaction of GSH with AP, the ring-opened isomer of 4-OOH-CP, to form hemithioacetal followed by ring-closure and hydroxylation to produce the

Figure 5 The influence of altering the initial GSH content of cells by prior L-BSO pretreatment of various duration on the subsequent kinetics of GSH depletion by 4-OOH-CP in two tumour cell lines. (a) SKOV-3 human ovarian cancer cell line, and (b) KHT murine sarcoma cell line. Inserts depict the GSH depletion kinetics by L-BSO alone. Individual symbols are the mean of triplicate determinations.

Figure 6 The relationship between the initial GSH content of cells and the rate of GSH depletion by 4-OOH-CP in two tumour cell lines. Concentrations of 4-OOH-CP used were 100 and 14.7 μM for the SKOV-3 and KHT tumour cell lines respectively. Error bars represent ± s.d. of three repeat experiments.

**Figure 7** (**a**) The effects of prior GSH depletion by L-BSO (4 h) on the intracellular alkylating capacity in SKOV-3 cells following 4-OOH-CP exposure (200 μM). Filled circles, controls; open circles, with L-BSO pretreatment. (**b**) The influence of GSH concentration on the alkylating capacity of 4-OOH-CP (200 μM in aqueous phosphate-buffered solution, pH 7.4. Alkylating capacity was determined by the 4-nitrobenzyl-pyridin (NBP)-test. Each symbol represents the average of triplicate determinations.

parent 4-OH-CP once again (Figure 1). This cyclic reaction has been well studied using $^{31}P$ NMR spectroscopy (Zon *et al.*, 1984; Kwon *et al.*, 1987). The effect of this reaction is the stabilisation of 4-OH-CP from spontaneous degradation to toxic metabolites (Figures 6a and 7b). The importance of GSH as a determinant of cellular sensitivity to cyclophosphamide and its activated metabolite, 4-OH-CP, is likely due to this two-tier mechanism.

Interestingly, the decelerating effect of GSH on the spontaneous degradation of 4-OH-CP appeared to be effective only at high GSH concentration. Thus, L-BSO was effective in enhancing the cytotoxicity and spontaneous degradation of 4-OH-CP for SKOV-3 cells, which have high GSH content, but was ineffective on both counts for KHT cells, which have low GSH content. This concentration dependent characteristic of GSH protection may have important implications regarding the use of L-BSO in combination with cyclophosphamide in cancer chemotherapy. It may be that this combination should optimally be used for resistant tumours with high GSH contents (Lee *et al.*, 1990), but may have little additional value, over treatment with cyclophosphamide alone, for sensitive tumours with low GSH content. Similarly, critical normal tissues, such as the bone marrow, containing lower GSH contents than tumours (Lee *et al.*, 1989) may be preferentially spared *vis-à-vis* tumours.

It should be noted that Draeger *et al.* (1976) has previously reported that GSH and other thiols could stabilize the alkylating capacity of 4-OH-CP by the reversible formation of 4-(alkylthio)cyclophosphamide. Zon *et al.* (1984) and Kwon *et al.* (1987) had subsequently confirmed this result using $^{31}P$ NMR spectroscopy. It was concluded that 4-OH-CP underwent slow ring opening to AP followed by facile formation of hemithioacetal, which then cyclised to 4-thiocyclophosphamide adducts (Kwon *et al.*, 1987). The present study extends these findings and, in addition, provides the first demonstration that the stabilising effects of GSH also occur within intact tumour cells. The toxification of 4-OH-CP takes place via the tautomer AP. GSH, via the futile cyclic reactions described above (Figure 1) shifts the tautomerism pseudoequilibrium between 4-OH-CP and AP in favour of the former tautomer. This has the effect of reducing the rate of formation of AC and PM, the 'ultimate toxic metabolite' (Connors *et al.*, 1974; Sladek, 1987).

The pivotal role of GSH was well illustrated in the companion study in which the chemosensitivity and GSH status of each of 17 tumour cell lines was determined. A significant direct correlation ($r = 0.84$, $P = 0.05$) was observed between GSH content and resistance to 4-OOH-CP (Lee *et al.*, 1990). It was also found that the GSH status of cells treated with 4-OOH-CP was an extremely effective predictor of chemosensitivity. The occurrence of a significant reduction of tumour cell reproductive capacity was always accompanied by substantial depletion of cellular GSH, and vice versa. The present model provides a ready explanation for the effectiveness of GSH status in predicting for cellular chemosensitivity to 4-OOH-CP. For a given cell line treated with a subtoxic dose of 4-OOH-CP, the stabilisation effect of GSH is sufficient to keep the formation of PM and AC to a minimum. Consequently the intracellular GSH content remains relatively unchanged following drug exposure. With increasing drug dose however, considerable amounts of toxic metabolites are formed. As a result (1) the intracellular GSH content begins to be depleted which will then further destabilise 4-OH-CP, and (2) the tautomerism pseudoequilibrium between 4-OH-CP and AP shifts progressively in favour of AP which will then further promote the formation of PM and AC. This vicious cycle of destabilisation and toxic metabolite formation is likely to rapidly lead to cell death.

It should be noted that in the formulation of the present model we have made two important assumptions: (1) The quantity of the ultimate alkylating metabolite (PM) formed during drug exposure is the dominant factor governing cellular chemosensitivity to 4-OH-CP. In this regard, it should be noted that factors others than GSH *per se* may also play significant roles in determining chemosensitivity by modulating PM formation. Two such factors are the enzymes aldehyde dehydrogenase and glutathione S-transferase. These enzymes have been shown to confer resistance to cyclophosphamide in some tumours and normal tissues (Sladek, 1987; Hilton, 1984; Carmichael *et al.*, 1986*a*; McGown & Fox, 1986). A cell line with higher activity of these enzymes may be able to detoxify AP more efficiently, thus minimising toxic metabolites formation (see Figure 1) and maintaining the GSH status quo. It is probably for this reason that monitoring the changes in GSH status appears to be better than monitoring absolute GSH content *per se* as a method of chemosensitivity prediction for 4-OOH-CP (Lee *et al.*, 1990). (2) It is also implicit in the proposed model that the *in vitro* thiol-reactive effects of 4-OH-CP and its metabolites can be extrapolated to *in vivo* conditions. Under some conditions, this may not necessarily be the case. It has been suggested that thiol-reactive agents might behave differently *in vivo* as compared to *in vitro* because of differences in the relative amount of thiols versus thiol-reactive agents present under the two conditions (Wardman & Clarke, 1987). For two reasons, however, it is believed that *in vitro–in vivo* extrapolation is justified for 4-OH-CP and its metabolites. Firstly, cyclophosphamide administration *in vivo* has been shown to cause GSH depletion *in vivo* (Adams *et al.*, 1986; Carmichael *et al.*, 1986*a*). Secondly, the important reversible equilibria in the proposed reaction scheme are probably adequately modelled *in vitro*, since relevant concentrations of the thiol-reactive agent have been used.

The proposed model also defines the precise role of acrolein in the overall activity of 4-OH-CP. A number of puzzling observations have long fueled the debate as to which metabolites of CP are responsible for its cytotoxic activity (Sladek, 1987) and oncostatic specificity (Sladek, 1987; Hohorst *et al.*, 1976). Even though PM is now generally regarded as the ultimate alkylating metabolite responsible for the *in vivo* and *in vitro* cytotoxic activity of CP and 4-OH-CP (Brock, 1976; Struck *et al.*, 1975), the fact that its cytotoxic activity and oncostatic specificity is much inferior, on a molar basis, to CP and 4-OH-CP (Brock, 1976; Hohorst *et*

al., 1976; Alberts *et al.*, 1984) remains unexplained. Exhaustive investigation had uncovered no other metabolites of significant cytotoxicity (Sladex, 1987), or which were able to form inter-strand DNA crosslinks (Erickson *et al.*, 1980; Cairney *et al.*, 1984). The basis of these paradoxical findings has frequently been attributed to the presumption, never conclusively proven, that PM has poor cell membrane transport characteristics (Hohorst *et al.*, 1976; Alberts *et al.*, 1984). The presence results suggest another, though not mutually exclusive, explanation: 4-OH-CP possesses superior cytotoxic activity to PM because of its ability to generate not only PM but also acrolein which, though not cytotoxic itself, augments the cytotoxic activity of 4-OH-CP. The possibility of such a sensitising action has been suggested by Alarcon and Meienhofer (1971) for acrolein, but no supporting evidence has been presented. In agreement with previous findings (Brock, 1976; Wrabetz *et al.*, 1980), we have also demonstrated the relative lack of toxicity of acrolein (Figure 4a). Thus, deschloro-4-hydroperoxycyclophosphamide, which has its *bis*-(β-chloroethyl)amine group replaced by diethylamine, produced acrolein (Alarcon & Meienhofer, 1971) and depleted cellular GSH as efficiently as 4-OOH-CP (Figure 4b), but was non-cytotoxic because it cannot give rise to PM (Figure 4a). The present model suggests that acrolein enhances the cytotoxicity of PM via the following sequence of events: (1) GSH depletion, (2) progressive destabilization of 4-OH-CP, and finally (3) enhancement of PM formation.

I wish to thank Dr D.W. Siemann for his support; the Cell Separation Facility of the University of Rochester Cancer Center for technical support; Dr R. Borch and Dr P. Hilgard for the supply of 4-hydroperoxycyclophosphamide and deschloro-4-hydroperoxycyclophosphamide; Dr R. Borch and Bruce Fenton for helpful comments; and D.J. Flannery for excellent technical assistance. Supported by PHS grant CA-38637.

### References

ADAMS, D.J., CARMICHAEL, J. & WOLF, C.R. (1986). Altered mouse bone marrow glutathione and glutathione transferase levels in response to cytotoxins. *Cancer Res.*, **45**, 1669.

ALARCON, R.A. & MEIENHOFER, J. (1971). Formation of the cytotoxic aldehyde acrolein during *in vitro* degradation of cyclophosphamide. *Nature, N. Biol.*, **233**, 250.

ALBERTS, D.S., EINSPAHR, J.G., STRUCK, R. & 4 others (1984). Comparative *in vitro* cytotoxicity of cyclophosphamide, its major active metabolites and the new oxazaphosphorine ASTA Z 7557 (INN mafosfamide). *Invest. N. Drugs*, **2**, 141.

ARRICK, B.A. & NATHAN, C.F. (1984). Glutathione as a determinant of therapeutic efficacy: a review. *Cancer Res.*, **44**, 4224.

BROCK, N. (1976). Comparative pharmacologic study *in vitro* and *in vivo* with cyclophosphamide (NSC-26271), cyclophosphamide metabolites, and plain nitrogen mustard compounds. *Cancer Treat. Rep.*, **60**, 301.

CAIRNEY, A.E., SLADEK, N.E. & WOODS, W.G. (1984). DNA crosslinking by 4-hydroperoxycyclophosphamide in sensitive and resistant mouse leukemia cells. *Proc. Am. Assoc. Cancer Res.*, **25**, 239.

CARMICHAEL, J., ADAMS, D.J., ANSEL, J. & WOLF, C.R. (1986*a*). Glutathione and glutathione transferase levels in mouse granulocytes following cyclophosphamide administration. *Cancer Res.*, **46**, 735.

CARMICHAEL, J., FRIEDMAN, N., TOCHNER, Z. & 4 others (1986*b*). Inhibition of the protective effect of cyclophosphamide by pretreatment with buthionine sulfoximine. *Int. J. Radiat. Oncol. Biol. Phys.*, **12**, 1191.

CONNORS, T.A., COX, P.J., FARMER, P.B., FOSTER, A.B. & JARMAN, M. (1974). Some studies of the active intermediates formed in the microsomal metabolism of cyclophosphamide and isophosphamide. *Biochem. Pharmacol.*, **23**, 115.

CROOK, T.R., SOUHAMI, R.L., WHYMAN, G.D. & MCLEAN, A.E.M. (1986). Glutathione depletion as a determinant of sensitivity of human leukemia cells to cyclophosphamide. *Cancer Res.*, **46**, 5035.

DRAEGER, U., PETER, G. & HOHORST, H.-J. (1976). Deactivation of cyclophosphamide (NSC-26271) metabolites by sulfhydryl compounds. *Cancer Treat. Rep.*, **60**, 335.

ERICKSON, L.C., ROMONAS, L.M., ZAHARKO, D.S. & KOHN, K.W. (1980). Cytotoxicity and DNA cross-linking activity of 4-sulfidocyclophosphamides in mouse leukemia cells *in vitro*. *Cancer Res.*, **40**, 4216.

FRIEDMAN, O.M. & BOGER, E. (1961). Colorimetric estimation of nitrogen mustards in aqueous media. Hydrolytic behavior of *bis*(beta-chloroethyl)amine, nor HN2. *Anal. Chem.*, **33**, 906.

GURTOO, H.L., HIPKENS, J.H. & SHARMA, S.D. (1981). Role of glutathione in the metabolism-dependent toxicity and chemotherapy of cyclophosphamide. *Cancer Res.*, **41**, 3584.

HILTON, J. (1984). Role of aldehyde dehydrogenase in cyclophosphamide-resistant L1210 leukemia. *Cancer Res.*, **44**, 5156.

HOHORST, H.-J., DRAEGER, U., PETER, G. & VOELCKER, G. (1976). The problem of oncostatic specificity of cyclophosphamide (NSC-26271): studies on reactions that control the alkylating and cytotoxic activity. *Cancer Treat. Rep.*, **60**, 309.

JOCELYN, P.C. (1972). *Biochemistry of the SH Group*. Academic Press: London.

KALLMAN, R.F., SILINI, G. & VAN PUTTEN, L.M. (1967). Factors influencing the quantitative estimation of the *in vivo* survival of cells from solid tumors. *J. Natl Cancer Inst.*, **39**, 539.

KOWN, CHUL-HOON, BORCH, R.F., ENGEL, J. & NIEMEYER, U. (1987). Activation mechanisms of Mafosfamide and the role of thiols in cyclophosphamide metabolism. *J. Med. Chem.*, **30**, 395.

LEE, F.Y.F., VESSEY, A.R. & SIEMANN, D.W. (1988). Glutathione as a determinant of cellular response to doxorubicin. *NCI Monogr.*, **6**, 211.

LEE, F.Y.F., VESSEY, A., ROFSTAD, E., SIEMANN, D.W. & SUTHERLAND, R.M. (1989). Heterogeneity of glutathione content in human ovarian cancer. *Cancer Res.*, **49**, 5244.

LEE, F.Y.F., SIEMAN, D.W. & FLANNERY, D.J. (1990). Prediction of tumor sensitivity to 4-hydroperoxycyclophosphamide by a glutathione-targeted assay. *Br. J. Cancer* (in the press).

MCGOWN, A.T. & FOX, B.W. (1986). A proposed mechanism of resistance to cyclophosphamide and phosphoramide mustard in a Yoshida cell line *in vitro*. *Cancer Chemother. Pharmacol.*, **17**, 223.

ONO, K. & SHRIEVE, D.C. (1987). Effects of glutathione depletion by L-buthionine sulfoximine on the cytotoxicity of cyclophosphamide in single and fractionated doses to EMT6/SF mouse tumors and bone marrow. *J. Natl Cancer Inst.*, **79**, 811.

RUSSO, A., CARMICHAEL, J., FRIEDMAN, N. & 4 others (1986). The roles of intracellular glutathione in antineoplastic chemotherapy. *Int. J. Radiat. Biol. Phys.*, **12**, 1347.

SIEMANN, D.W., LORD, E.M., KENG, P.C. & WHEELER, K.T. (1981). Cell subpopulations dispersed from solid tumors and separated by centrifugal elutriation. *Br. J. Cancer*, **44**, 100.

SIEMANN, D.W., FLAHERTY, A.A. & PENNEY, D.P. (1989). Effect of thiol manipulation on chemopotentiation of nitroimidazoles. *Int. J. Radiat. Oncol. Biol. Phys.*, **16**, 1341.

SLADEK, N.E. (1987). Oxazaphosphorines. In *Metabolism and Action of Anti-cancer Drugs*, Powis, G. & Prough, R.A. (eds), p. 48. Taylor and Francis: London.

STRUCK, R.F., KIRK, M.C., WITT, M.H. & LASTER, W.R. (1975). Isolation and mass spectral identification of blood metabolites of cyclophosphamide: evidence for phosphoramide mustard as the biologically active metabolite. *Biomed. Mass. Spectrometry*, **2**, 46.

WARDMAN, P. & CLARK, E.D. (1987). Redox properties and rate constants in free-radical mediated damage. *Br. J. Cancer*, **55** (suppl. VIII), 172.

WHILLANS, D.W. & RAUTH, A.M. (1980). An experimental and analytical study of oxygen depletion in stirred cell suspensions. *Radiat. Res.*, **84**, 97.

WRABETZ, E., PETER, G. & HOHORST, H.-J. (1980). Does acrolein contribute to the cytotoxicity of cyclophosphamide? *J. Cancer Res. Clin. Oncol.*, **98**, 119.

ZON, G., LUDEMAN, S.M., BRANDT, J.A. & 4 others (1984). NMR spectroscopic studies of intermediary metabolites of cyclophosphamide. A comprehensive analysis of the interconversion of *cis* and *trans*-4-hydroxycyclo-phosphamide with aldophosphamide and the concomitant partitioning of aldophosphamide between irreversible fragmentation and reversible conjugation pathways. *J. Med. Chem.*, **27**, 466.